# Keeping α-Synuclein at Bay: A More Active Role of Molecular Chaperones in Preventing Mitochondrial Interactions and Transition to Pathological States?

**DOI:** 10.3390/life10110289

**Published:** 2020-11-19

**Authors:** Emelie E. Aspholm, Irena Matečko-Burmann, Björn M. Burmann

**Affiliations:** 1 Department of Chemistry and Molecular Biology, University of Gothenburg, 40530 Göteborg, Sweden; emelie.aspholm@gu.se; 2 Wallenberg Centre for Molecular and Translational Medicine, University of Gothenburg, 40530 Göteborg, Sweden; irena.burmann@gu.se; 3 Department of Psychiatry and Neurochemistry, University of Gothenburg, 40530 Göteborg, Sweden

**Keywords:** Parkinson’ disease, α-synuclein, post-translational modifications, molecular chaperones, mitochondria, mitochondrial proteases

## Abstract

The property of molecular chaperones to dissolve protein aggregates of Parkinson-related α-synuclein has been known for some time. Recent findings point to an even more active role of molecular chaperones preventing the transformation of α-synuclein into pathological states subsequently leading to the formation of Lewy bodies, intracellular inclusions containing protein aggregates as well as broken organelles found in the brains of Parkinson’s patients. In parallel, a short motif around Tyr39 was identified as being crucial for the aggregation of α-synuclein. Interestingly, this region is also one of the main segments in contact with a diverse pool of molecular chaperones. Further, it could be shown that the inhibition of the chaperone:α-synuclein interaction leads to a binding of α-synuclein to mitochondria, which could also be shown to lead to mitochondrial membrane disruption as well as the possible proteolytic processing of α-synuclein by mitochondrial proteases. Here, we will review the current knowledge on the role of molecular chaperones in the regulation of physiological functions as well as the direct consequences of impairing these interactions—i.e., leading to enhanced mitochondrial interaction and consequential mitochondrial breakage, which might mark the initial stages of the structural transition of α-synuclein towards its pathological states.

## 1. Introduction

The pathological hallmark for Parkinson’s disease and related synucleinopathies is the accumulation of the pre-synaptic protein α-synuclein (product of the *SNCA* gene) in Lewy body aggregates within the brain together with the degeneration of dopaminergic neurons within the *substantia nigra compacta* [1,2]. Although ageing as well as cellular oxidative stress are known to be common factors driving synucleinopathy progression [3,4], Lewy body morphologies as well as α-synuclein aggregates or fibrillar structures differ in a disease-specific manner. These different diseases are either based on gene duplications of the *SNCA* gene, in the case of inherited forms of Parkinson’s disease, or missense mutations within the first ~55 residues of the *SNCA* gene in multiple systems atrophy (MSA) and dementia with Lewy bodies (DLB) [5,6,7,8,9,10,11,12,13,14,15]. These observations indicate that fibril formation and aggregate compositions are likely modulated by context-dependent cellular factors, such as mutations and/or post-translational modifications, and are so far poorly understood [16]. Furthermore, the toxicity and spreading of different α-synuclein oligomers and aggregates vary for different brain regions and cellular types [17], highlighting the underlying heterogeneity of α-synuclein aggregate formations within the different synucleinopathies [18].

Although the ability of molecular chaperones to dissolve protein aggregates of α-synuclein was identified as a possible important cellular checkpoint altering the pathological structural adaptions of α-synuclein, its structural details together with a possible generality of this effect remained elusive [19,20,21,22]. Among the identified chaperones were the most abundant cytosolic molecular chaperones of the Hsp70 [19,21,23] and Hsp90 [24,25,26] families together with members of the small heat shock proteins (sHSPs) [27,28,29] as well as mitochondrial chaperones, interestingly (e.g., Hsp10, TRAP1) [30,31], and metal-dependent chaperones [32,33]. In recent years, several detailed analyses of a large pool of molecular chaperones elucidated the generality of this interplay, even pointing to a more active role of a diverse set of molecular chaperones by regulating the physiological function of α-synuclein, therefore preventing the transformation of α-synuclein towards pathological states [24,29,32,34]. These initial pathological states play a key role in the subsequent formation of Lewy bodies, cellular inclusions containing protein aggregates as well as broken organelles, which are found in the brains of Parkinson’s patients [6,18,35]. In the context of these detailed characterizations, two short motifs at the amino-terminus as well as centered around Tyr39 could be identified to be a crucial mediator of the aggregation propensity of α-synuclein [34,36,37,38]. Remarkably, these two regions also comprise the main segments in contact with a diverse pool of molecular chaperones [32,34]. Furthermore, it could be shown that the inhibition of the chaperone:α-synuclein interaction facilitates binding of α-synuclein to mitochondria in combination with α-synuclein aggregation [34]. Despite the existence of functional clearance systems, such as the autophagy, proteasomal as well as lysosomal systems for cytoplasmic α-synuclein, the reason for the impaired clearance remains unknown, though alterations in post-translational modification patterns [39,40,41] might play a role in the observed relocalization from lysosomes to mitochondria upon chaperone inhibition [34]. This subcellular relocalization of α-synuclein might facilitate mitochondrial membrane disruption [42,43,44], indicating the possible likelihood of proteolytic processing of α-synuclein by mitochondrial proteases [5,34,35,45]. Herein, we will review the current knowledge on the role of molecular chaperones in the regulation of the physiological function as well as the direct consequences of impairing these interactions, which might mark crucial aspects of the initial stages of the structural transition of α-synuclein towards its pathological states.

## 2. Chaperone: α-Synuclein Interplay

**Figure 1:**
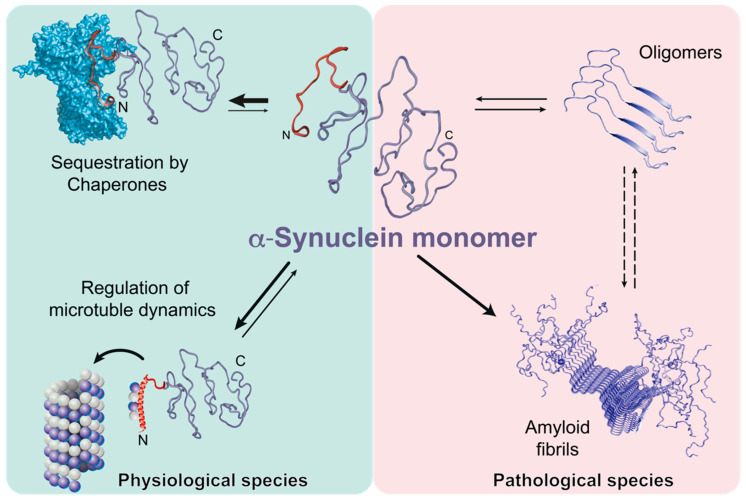
Mechanism of chaperone-controlled regulation of α-synuclein function in mammalian cells. Molecular chaperones (blue, e.g., Hsp70s, Hsp90s, small HSPs) interact with the ~40 amino-terminal residues of α-synuclein, also including crucial Tyr39 (red), thus actively regulating its functional species by shifting conformational equilibria, and therefore actively preventing the transitions towards pathological states.

Initially, some molecular chaperones were shown to have the ability to dissolve existing α-synuclein fibrils in an ATP-dependent manner in vitro [19] by modulating fibrillization kinetics [21]. Despite the high interest in the prevention of α-synuclein fibrillization by molecular chaperones, the structural basis, the functional consequences, and the generality of chaperone:α-synuclein interactions remained unknown for a long time [20]. A recent study provided a more comprehensive and novel insight into the role of molecular chaperones [34]. A large pool of structurally different test chaperones as well as relevant mammalian chaperones was used to map the interacting regions of α-synuclein revealing the amino-terminus as well as the region surrounding Tyr39 as the common chaperone binding site [32,34]. The observed effects for the two most abundant mammalian chaperones, Hsp90β and Hsc70, indicated an extended binding interface on Hsp90β [34,46] and an ATP-dependent interaction of Hsc70 [34] due to the reported interaction mode of Hsc70 with the fibrillar α-synuclein for disassembling these into monomers/oligomers [19,21]. Taken together, this distinct binding site on α-synuclein was the first indication of a direct involvement of molecular chaperones in regulating the physiological role of α-synuclein—e.g., synaptic budding events [47,48] as well as microtubuli dynamics [49,50,51,52]—whereas the exact role of α-synuclein either as a direct interactor with microtubuli and/or facilitator of microtubuli:Tau interactions remains to be discerned [50]. Therefore, by regulating the amount of free α-synuclein in the neuronal cell chaperones actively prevent its transition towards the pathological states (Figure 1).

To obtain a detailed insight into the chaperone interactome of the α-synuclein amino-terminus within mammalian cells, a cross-linking mass-spectrometric analysis of wild-type α-synuclein and ∆N-α-synuclein, a variant lacking parts of the crucial amino-terminus [37], was performed [34]. The resulting interactome revealed that a large diversity of different chaperones from the Hsp90 and Hsp70 family interacted with the amino-terminus of α-synuclein. Furthermore, different foldase machineries were found to be interacting with α-synuclein: the mammalian mitochondrial Hsp60 chaperone as well as seven out of the eight different subunits of the hetero-oligomeric cytosolic TRiC/CCT-chaperonin, possibly due to the presence of large hydrophobic patches within the substrate recognition sites of the chaperonin subunits [53].

As one of the known roles of the amino-terminal region of α-synuclein is its interaction with cellular membranes [54,55], displacement titrations were used showing that the chaperone interactions dominate the membrane interaction [34]. The observation that α-synuclein binding to chaperones can dominate its vesicle interaction was also independently shown in earlier studies for Hsp90β [24] and Hsp27 [29].

Investigating the role of molecular chaperones by sophisticated in-cell NMR spectroscopy in living mammalian cells [34,56,57] enabled us to directly study the effect of chaperone inhibition in the cellular context. Inhibiting the two main molecular chaperones, Hsc70 and Hsp90, indicated that the release of α-synuclein leads to a direct interaction with cellular membranes, as evident from the characteristic interaction pattern for the first 100 residues [34,56,58]. Subsequent immunofluorescence analysis showed that, upon chaperone depletion, α-synuclein directly associates with the mitochondria [34], whose possible implications will be discussed in detail below. Besides this interaction on the amino-terminus, in-cell NMR also revealed specific interactions at the carboxy-terminus [34,56], where a recent pre-print by the Knowles and Hartl labs points to an Hsp40 chaperone, DnaJB1, that interacts with α-synuclein in this region [59], presumably providing another layer of control for the amount of free α-synuclein—awaiting further verification in the future.

## 3. Importance of the α-Synuclein Amino-terminus

Interestingly, within mammalian cells, α-synuclein predominantly exists as an amino-terminally acetylated form as the physiologically relevant species [56,60]. Structurally, this post-translational modification leads to an increased helical propensity within the α-synuclein amino-terminus [58,61]. Although not being part of the fibrillar core of the ensuing α-synuclein fibril [8,62], very recently the importance of this region in promoting α-synuclein aggregation could be established [36,37,38]. By binding to this region, molecular chaperones therefore reduce the amount of free α-synuclein in the cells and thus actively prevent the transition towards pathological states [32,34]. Therefore, as a consequence, amino-terminal binding might regulate the exposure of the non-amyloidic core (NAC) region of α-synuclein [63,64], containing the central element of the α-synuclein-fibrils [62]. Remarkably, the exposure of this crucial amino-terminal region can also be modulated through different carboxy-terminal interactions indicating intramolecular communication between its termini despite the absence of any structural elements [38]. On the one hand, it could be shown that Ca^2+^ binding to carboxy-terminal residues [65,66] as well as proteolytic cleavage in the same region [67] resulted in enhanced aggregation propensity, in line with the proposed importance of the amino-terminal segment governing the transition to fibrillar states [36]. In particular, the Ca^2+^:α-synuclein interaction is physiologically highly relevant (see next section) as it could be shown that calcium buffering becomes dysregulated in Parkinson’s disease and that an increase in cytosolic calcium is observable [68].

## 4. α-Synuclein and Mitochondrial Membranes: A Fatal Relationship?

Under normal cellular conditions, α-synuclein is primarily a cytosolic protein showing no obvious enrichment on cellular and mitochondrial membranes [69,70], but ultrastructural studies clearly showed the direct interaction between mitochondria and α-synuclein in particular within dopaminergic neurons [71,72], and, in addition, a general affinity for different cellular membranes within the neuronal synapse [73]. Furthermore, Lewy bodies have lately been characterized in detail revealing their content as a medley of proteins and broken organelles including mitochondria [5,6,18]. 

Strikingly, most interactions with mitochondria are observed in cells exposed to enhanced oxidative stress [3], higher protein expression [74], or an impaired chaperone:α-synuclein ratio [34], clearly pointing to impairments in protein clearance mechanisms, whose exact nature so far remain largely elusive [40,41,75]. Remarkably, in cells with increased α-synuclein expression, the protein accumulates at and within inner mitochondrial membranes while normally it is mainly attached to the outer membranes [71,74,76,77]. Notably, in patients with Parkinson’s disease, a significant amount of α-synuclein was observed to be localized within the inner membranes [78]. However, given the well-established pre-synaptic localization of α-synuclein and its role in synaptic vesicle release [79], it remains unclear how much physiological α-synuclein is mitochondria-associated and how it exactly affects mitochondrial function. One possible native cellular function is the regulation of the mitochondrial ATP synthase machinery [49,80], whose possible role was further indicated by cross-linking mass spectrometry indicating a direct interaction with the amino-terminus of α-synuclein and components of this cellular machinery embedded in mitochondria [34].

**Figure 2:**
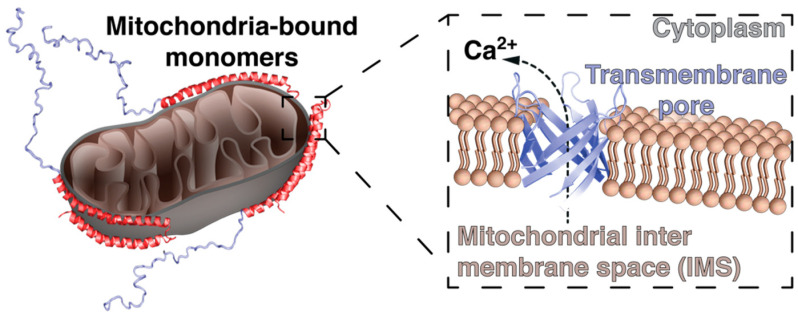
Possible initial consequences of an enhanced mitochondrial interaction of α-synuclein, possibly leading to mitochondrial membrane rupture in conjunction with the efflux of ions, which subsequently impairs cellular function by, e.g., overactivating Ca^2+^-dependent cellular pathways and impairing the cellular clearance mechanism.

Several studies have shown the membrane-associated folding of the protein that underlines dynamics that affect protein homeostasis in vivo [79]. To understand the disease pathway, it is particularly important to understand all the factors that trigger the protein misfolding and their role in pathophysiology. Several studies have shown that the α-synuclein aggregation depends on the dynamic equilibrium between the native structures [60,81,82,83]. The protein is natively unfolded and can adopt different conformations depending on the interactions within the cell. There are several factors that control aggregation of α-synuclein such as high expression due to gene mutations, acidic conditions (observed in patients with Parkinson’s disease), and influence of the interplay with cellular metabolites [84,85]. Furthermore, the amino-terminal region of α-synuclein can also bear several different post-translational modifications (PTMs), such as tyrosine-phosphorylation [86,87,88], methionine oxidation [89,90], non-enzymatical glycation [91,92], and sumoylation [93,94] directly affecting its membrane binding propensity as well as modulating its ability to aggregate. The analysis of the primary structure of the protein and its features in the cell under physiological conditions suggests a membrane-mediated homeostatic function of the protein in vivo [95]. α-Synuclein harbors diverse affinities for different membranes adopting alternate conformations, which possibly underlines its homeostatic function at the lipid–peptide interface [44,55,73,79]. Depending on the lipid interface composition, the α-synuclein conformation might acquire the potential to disrupt the associated membrane [96], or promote cellular signaling—e.g., through ion release upon mitochondrial disruption [97] (Figure 2). 

α-Synuclein possesses the inherent ability to interact with a diversity of membrane surfaces [73] within the cellular context but it shows a strong affinity towards mitochondrial membranes [34,98]. The underlying reason could be the composition of the mitochondrial membranes and in particular the presence of cardiolipin [98,99,100]. The presence of cardiolipin within the lumen of the mitochondrial membranes generates a negative charge on the membrane surface causing the molecular affinity to α-synuclein, likely modulated via positively charged lysine residues in α-synuclein, and in line with in vitro observation of its preference for negatively charged lipids [55,95,101]. If this specific molecular attraction, which also might be accompanied by a refolding of α-synuclein, is associated with its mitochondria-associated natural function or might rather be a pre-state to, presumably, pathological mitochondrial membrane pores formed by α-synuclein leading subsequently to calcium (Ca^2+^) efflux [42,102,103], remains to be clarified in the future. Under normal physiological conditions cardiolipin is mainly found in the inner mitochondrial membrane, but it has been shown that under stress conditions it can be translocated to the outer mitochondrial membrane [104,105,106]. This translocation is one of the signals initiating mitophagy, the cellular process of degrading non-functional mitochondria via the autophagy process [107,108]. Intriguingly, the observation of broken mitochondria and non-functional autophagosomes within Lewy bodies [6,18,35] points to a possible role of α-synuclein in the impairment of this recycling machinery [109,110], a process which is so far only poorly understood. α-Synuclein can be degraded by the ubiquitin-proteasome system (UPS) as well as by macroautophagy, and impaired function in one system can be compensated by the other system [40,41,111,112]. However, larger protein aggregates can fail to be degraded by the proteasome [113]. Autophagy might therefore be the principal mechanism by which larger protein aggregates are cleared, in particular in the nervous system [114]. Wild-type α-synuclein has been shown to impair macroautophagy when overexpressed, providing a possible explanation as to why α-synuclein might in some cases not be able to be efficiently cleared by the cell [115]. Although, there is also an alternative method of clearance for α-synuclein—chaperone-mediated autophagy (CMA), which relies on the recognition of α-synuclein by the major chaperone Hsc70. It could be shown that mutations in α-synuclein itself impair this route [75]. In addition, the requirement of Hsc70 makes this system dependent on the chaperone levels in the neuronal cells, which already could be shown to be declining with age [116,117,118] and the chaperone is also a key factor for controlling the amount of free α-synuclein in the cells [34], therefore interfering with the CMA clearance pathway [119].

In addition to electrostatic attraction between α-synuclein and cardiolipin, other factors seem to play an important role in the binding of α-synuclein to mitochondria and subsequent pore-formation. Membrane curvature is a significant factor for the initialization of the mitochondrial fission and fusion processes. The membrane curvature in compliance with the association of α-synuclein might serve to enhance the fragmentation effects [42,120,121]. The dimeric structure of cardiolipin with its small head group and big hydrophobic tail help the membrane to adopt a conical shape [122]. This molecular shape also affects the package of the lipids and membrane stability.

The amino-terminal region of α-synuclein contains 11-amino acid (KTKEGVVAAAE) repeat sequences, allowing the formation of α-helices upon membrane interaction [44,54,55,58], reflecting the proteins’ capability to bind phospholipid vesicles [123]. Several studies have shown that the amino-terminal region, due to a large amount of positively charged lysines, is a mediator in anchoring to the membrane surface. The interaction can be explained as an electrostatic interaction between positively charged lysines and negatively charged cardiolipin. α-Synuclein forms an amphipathic helix upon membrane binding [124] followed by the insertion of the amphipathic helix directly into the lipid bilayer, causing membrane stress [125]. After this initial attachment to the membrane, the molecule stabilizes its hydrophobically central core, making it subsequently prone to aggregation and fibrillization [54,63,124,126].

Due to its unfolded native structure there are numerous possibilities for α-synuclein to translocate through the mitochondrial matrix under physiological conditions either through the standard protein-import machinery consisting of the translocase proteins of the outer membrane (TOM) and inner membrane (TIM) [127,128,129] or by passing through the most abundant outer membrane protein, the voltage gated anion channel (VDAC) [130,131]. Under these conditions, α-synuclein likely interacts with the ATP synthase subunit α and therefore plays an important role in controlling mitochondrial metabolism and maintaining the bioenergetic needs of the neuronal synapse [80,132,133,134]. In this context, the observation of interactions between mitochondrial molecular chaperones, such as Hsp10 [31], Hsp60 [34] and TRAP1 [30], and α-synuclein might indicate a regulative role of these chaperones in the mitochondrial context, which awaits further clarification in the future.

α-Synuclein’s interaction with TOM20 inhibits its interaction with co-receptor TOM22 which inhibits the mitochondrial protein-import machinery [135] and causes a reduced mitochondrial membrane potential, enhancing the production of reactive oxygen species (ROS). In addition, the outlined uncontrolled interaction with mitochondrial membranes might lead to a break-down of this fine-tuned interplay leading to the formation of α-synuclein pores in the mitochondrial outer membrane contributing directly to mitochondrial dysfunction [102] (Figure 2).

Clinical tests with Parkinson’s disease patients have shown that α-synuclein can interact with the mitochondrial complex-1 resulting in its reduced activity [78] which increases ROS production, proton leakage and decreases the maximum oxidative phosphorylation capacity [136]. The formation of ROS from mitochondria plays a significant role in the degeneration of neuronal cells [137]. Mitochondrial impairment due to overexpression of α-synuclein has been shown to generate excessive ROS that may either cause alteration of signal transduction [138] or result in genomic instability [139]. Oxidative stress conditions have been shown to facilitate aggregation of α-synuclein directly associated with mitochondrial dysfunction [89,140]. It is worth mentioning that the presence of iron and hydrogen peroxide accelerated the aggregation of α-synuclein in vitro [85,141], suggesting an increased modification of α-synuclein coupled to increasing ROS. In the presence of superoxide dismutase (SOD), unreacted superoxide is converted into the reactive and stable free radical hydrogen peroxide which has high potential in terms of membrane permeability and can oxidize iron–sulphur cluster-containing proteins [142]. 

Overexpression of ROS in the presence of nitric oxide (NO) can also lead to the production of the potent oxidant and nitrating agent peroxynitrite (ONO2-) and consequently, other reactive nitrogen species (RNS). The aggregation of α-synuclein has also been shown to be associated with increased oxidative or nitrosative stress [143,144]. Nitrated α-synuclein has an increased tendency to form dimers and oligomers by making cross-links between two tyrosine residues [145,146]. The latest findings point to the fact that this oxidative α-synuclein aggregation scavenges cytochrome c activity, thereby inhibiting the activity of this pro-apoptopic messenger and thus delaying the onset of programmed cell death [145,147].

The effect of α-synuclein on the respiratory chain system has also been monitored using different inhibitors that helped to evaluate the consequences of mitochondrial dysfunction on the aggregation kinetics of the protein [148,149,150]. The results obtained from different experimental systems, including in vitro, cell cultures and transgenic mice, strongly suggest that mitochondrial dysfunction and oxidative stress may cause α-synuclein aggregation.

In the mammalian cell, the known familial amino-terminal mutations, such as A30P and T6K, in α-synuclein have been shown to inhibit both mitochondrial fragmentation as well as mitochondrial morphology interactions in vivo [74]. The effect of α-synuclein on mitochondrial morphology involves an increase in fission rather than a block in fusion [76]. Interestingly, α-synuclein oligomers had a greater significant impact on mitochondrial fission as opposed to the monomeric forms. This observation further suggests that the protein binding is not solely sufficient to induce fragmentation, but the formation of oligomers and/or membrane pores exert the toxicity [77].

## 5. α-Synuclein Processing by Mitochondrial Proteins: A Facilitator of Parkinson’s Disease?

The transition of α-synuclein to mitochondria, as discussed in detail in the previous section, alters its subcellular localization and therefore also changes the pool of proteins that are interacting with α-synuclein either under physiological or pathological conditions. Mitochondria house a myriad of proteases, many of which have been known for a long time to be involved in the mitochondrial stress response and protein quality control machinery [151]. These proteases contribute to the overall health of the cell by maintaining the fitness of the mitochondria in a multitude of ways, such as through the removal of damaged and misfolded proteins, protection against oxidative damage, as well as by regulating mitophagy, the controlled degradation of damaged depolarized mitochondria [107]. Several mitochondrial proteases have been implicated in neurological diseases as loss-of-function mutations have been identified within patients suffering from neurological disease, but also through studies showing that mitoproteases interact with and affect other proteins of known importance in the progression of neurological diseases [151,152,153,154]. Studies have indicated that there are direct connections between the dysfunction of mitoproteases and abnormal aggregation of α-synuclein, leading to synucleinopathy progression (94–96). In the following section we will discuss the current knowledge of the involvement of mitoproteases in the progression of synucleinopathies in light of the association of α-synuclein with mitochondria. 

DJ-1 (*PARK7*) is a cellular protease translocated from the cytoplasm to the mitochondria under oxidative stress conditions [155,156]. On a functional site, it could be shown that DJ-1 is able to interact with both monomeric and oligomeric α-synuclein species reducing the propensity of α-synuclein oligomerization [157]. Mutations within DJ-1, associated with Parkinson’s disease, reduce the capacity of DJ-1 to interact with α-synuclein and its ability to reduce α-synuclein dimerization as well as toxicity. This indicates that DJ-1 is important in the prevention of the initial α-synuclein aggregation steps [158,159,160]. The ability of DJ-1 to effectively inhibit α-synuclein aggregation appears to be dependent on the oxidation state of its Cys106 residue [157,161,162]. The importance of DJ-1 to function as an important cellular checkpoint controlling the amount of α-synuclein in proximity of mitochondria is also reflected by the fact that DJ-1 has been found in the proximity of Lewy bodies [163,164], indicating that under these pathological conditions the house-keeping role of DJ-1 is overwhelmed in synucleopathies [6,18].

High temperature requirement protein A2 (HtrA2) is a serine protease localized within the inner membrane space (IMS) of the mitochondrion [165]. Current knowledge about its functional cycle indicates that HtrA2 carries out comparable functions, as its bacterial homologs DegP, DegS and DegQ, in the recognition and subsequent proteolytic cleavage of oxidatively damaged and misfolded proteins by recognizing exposed hydrophobic patches through its carboxy-terminal substrate recognition domain [166,167]. A possible neuroprotective role of HtrA2 has already been demonstrated experimentally. Mice carrying a loss-of-function variant of HtrA2 were shown to develop Parkinson-like symptoms including neurodegeneration and mitochondrial degeneration. In addition, identified mutations in the *HTRA2* gene cause hereditary tremors in humans which can progress into Parkinson’s disease [168,169,170]. Furthermore, HtrA2 has been found to co-localize with α-synuclein within Lewy bodies, and it could be shown that HtrA2 can reduce the propensity of α-synuclein seeding while also aiding the removal of already aggregated α-synuclein [45,171,172]. In addition, the proteolytic activity of HtrA2 is affected by PINK1, another protein whose dysfunction is linked to Parkinson’s disease. PINK1 has been shown to regulate the phosphorylation state of HtrA2 at S142 in a p38 stress pathway-dependent manner [173,174,175], possibly either by itself directly phosphorylating HtrA2 or alternatively by facilitating HtrA2 phosphorylation by other kinases [175]. In patients with idiopathic Parkinson’s disease, phosphorylation of HtrA2 at Ser142 is increased, and patients with Parkinson’s disease and mutations in the PINK1 gene have been shown to have diminished levels of Ser142-phosphorylated HtrA2 [174]. HtrA2 and PINK1 might thus both participate in a response which aims to protect the cell against mitochondrial stress [173]. 

Another evolutionally conserved mitochondrial protease is the hexameric Lon protease, which plays an important role in clearing oxidatively damaged proteins in mitochondria and which has been found in high concentrations in the *substantia nigra* of patients with Parkinson’s disease. It has also demonstrated an ability to reduce the aggregation propensity of α-synuclein [45]. Specifically, inhibiting Lon protease with small molecule inhibitors led to an attenuation of α-synuclein aggregation in a cell culture model [45,176].

ClpP, the proteolytic unit of the ClpXP chaperone–protease complex, is involved in mitochondrial protein turnover as well as degradation of misfolded and damaged protein in mitochondria [177]. Recently, it was shown that wild-type α-synuclein in Parkinson models and in the brains of Parkinson’s patients interacts with ClpP, causing reduced ClpP levels by promoting ClpP aggregation as well as a reduction in the proteolytic activity of ClpP [176]. The α-synuclein mutant A53T, linked to early on-set Parkinson’s disease [178], is even more prone to co-aggregate with ClpP than the wild-type α-synuclein, and has a more severe impact on the proteolytic capacity of ClpP [176]. Reduced levels of ClpP cause an increase in misfolded mitochondrial proteins and of oxidative damage to the mitochondria [176]. 

Although the mitochondrial proteases discussed so far antagonize α-synuclein accumulation and aggregation, studies have found evidence of truncated species of α-synuclein being present in the brains of patients with Parkinson’s disease [179,180], highlighting the possibility of overwhelming this protective mechanism. Furthermore, in vitro evidence points towards enhanced aggregation of carboxy-terminal cleaved α-synuclein by pro-apoptotic cytosolic caspases [67], indicating the possibility that unwarranted proteolytic cleavage could also can have unwanted side-effects. A candidate mitochondrial protease for the processing of α-synuclein into these truncated species is the calcium-regulated calpain protease, which has been found to have an increased activity within the *substantia nigra* of patients with Parkinson’s disease [181,182]. Calpains, in particular calpain-1, cleave α-synuclein and these truncated forms of α-synuclein are prone to forming high-molecular weight species of aggregated α-synuclein [182]. Calpain can produce both amino-terminally and carboxy-terminally truncated forms of wild-type α-synuclein as well as of the aggregation-prone α-synuclein familial mutant A30P [183]. Cleaving of α-synuclein by calpain induces a structural change from a random coil to a β-sheet structure, which causes α-synuclein to be more prone to aggregate [182,183,184]. An in vitro study suggested that fragments resulting from calpain processing of soluble α-synuclein prevented fibrillization of wild-type and A53T α-synuclein, while calpain processing of fibrillar α-synuclein induced the formation of insoluble α-synuclein aggregates [185]. This observation might point to the formation of alternate β-sheet rich structures such as the α-synuclein pores suggested to be formed in the mitochondrial membranes as discussed above [42,43,79].

## 6. Conclusions

In conclusion the finding that molecular chaperones are in constant contact with α-synuclein in the cellular context [32,34] by restricting the flexibility and accessibility of the amino-terminal region opens new perspectives for eventual pharmaceutical intervention [34]. Especially in light of the proposed crucial role of the amino-terminal segment in the structural transition of α-synuclein [36,38,186], these recent studies indicate possibly early events in the transitions directly leading to α-synuclein mitochondrial interactions [34]. In this context, the importance of Tyr39 might be an important feature for future studies of pharmacological intervention, as phosphorylation of this residue by oxidative stress-induced Abelson kinase [86,187,188] impairs the protective chaperone interaction [34]. The potential of this route has already started to be investigated by addressing the use of Abelson kinase inhibitors [189].

Based on the observation of a large amount of broken mitochondria and the mitochondria recycling machinery within Lewy bodies [5,6,18], a possible intermediate stage with α-synuclein partially destroying mitochondria in neuronal cells [42,190] might mark crucial steps in the early stages of the disease. Nevertheless, further research on the early stages of the disease’s progression is needed to be able to identify the underlying details in sufficient detail to develop effective drugs impairing or delaying this neurodegenerative process.
